# The Prevalence and Associations of Peripheral Retinopathy: Baseline Study of Guangzhou Office Computer Workers

**DOI:** 10.1155/2018/2358690

**Published:** 2018-06-20

**Authors:** Ting Zhang, Yajing Zuo, Yantao Wei, Wenbin Huang, Xuezhi Zhou, Rongjiao Liu, Lili Zhong, Manjuan Peng, Shaochong Zhang

**Affiliations:** ^1^State Key Laboratory of Ophthalmology, Zhongshan Ophthalmic Center, Sun Yat-Sen University, Guangzhou, China; ^2^Department of Ophthalmology, The Second Affiliated Hospital of Guangzhou Medical University, Guangzhou, China

## Abstract

**Purpose:**

To determine the prevalence of peripheral retinopathy and its associated risk factors among a sample of Guangzhou office computer workers.

**Methods:**

A cross-sectional study of Guangzhou Chinese computer workstations and operators in different departments and units of the Guangzhou Power Supply Bureau, China, in 2016. Peripheral retinopathy was recorded and analyzed using a scanning laser ophthalmoscope (SLO; Optos, Daytona, United Kingdom) and slit-lamp microscopy combined with a three-mirror contact lens.

**Results:**

The 1934 eyes of 967 subjects (513 females and 454 males) were included in this study. In total, 79.1% of the eyes were myopic in workers aged 20–29 years, 72.9% in workers aged 30–39 years, 62.2% in workers aged 40–49 years, and 43.4% in workers aged 50–59 years (*p* < 0.001). Most eyes had optic nerve crescents (81.3%). Various peripheral degenerations were found: 7 eyes (0.4%) had microcystoid degeneration, 40 (2.1%) had peripheral pigmentary degeneration, 87 (4.5%) had lattice degeneration, and 4 (0.2%) had snail-track degeneration. Nineteen (1.0%) eyes had paving-stone degeneration, 11 (0.6%) eyes had a retinal hole or tear, and 16 (0.8%) eyes had chorioretinal degeneration. Multivariate regression confirmed that greater axial length (OR: 1.18 (1.03, 1.35), *p*=0.012) and more serious spherical equivalent (OR: 0.82 (0.77, 0.88), *p* < 0.001) were significant risk factors for peripheral retinal changes.

**Conclusion:**

Peripheral retinal degenerative changes were found in a larger proportion of younger computer workers than older ones. Myopia is occurring in younger and younger people, accompanied by peripheral retinal degeneration.

## 1. Introduction

The dramatic rise in computer use during the last few decades has raised concerns about the potentially deleterious health effects of increased “screen time” and associated short-wavelength (blue) light exposure. Increasing numbers of individuals are reportedly experiencing varieties of “computer vision syndrome” (CVS) [1, 2], which includes irritated and dry eyes, eye strain/fatigue, red eyes, blurred/double vision, burning eyes, excessive tearing, light/glare sensitivity, headaches, slowness in changing focus, and changes in color perception [3, 4]. However, the possibility that retinal changes occur in office computer workers remains to be confirmed. The blue component in computer screen illumination is relatively strong, but it is not nearly intense enough to produce acute retinal damage. Nevertheless, the potential exists for long-term, cumulative effects, including cellular damage arising primarily from increased oxidative stress [5, 6].

The tremendous use of computers by staff members, technicians, and students at King Abdulaziz University (KAU), revealed by data from a previous study, has been accompanied by an increase in the number of visits to the University Medical Directorate (Services) for eye and vision complaints [7]. Thus, Chinese office computer workers are also likely to have a significant prevalence of CVS, with its associated loss of productivity and compromised quality of life. The aim of the present study is to describe the prevalence of peripheral retinopathy and its associated risk factors among a representative sample of Guangzhou office computer workers who operate the 976 computer workstations in the 32 different departments and units of the Guangzhou Power Supply Bureau, Guangdong Province, China.

The study seeks to determine the prevalence and risk factors of computer use-related retinal changes in office workers, to provide useful information for the public on this ocular health issue, and to raise awareness about these issues among eyecare personnel.

## 2. Patients and Methods

### 2.1. Study Population

This was a population-based cross-sectional study conducted in Guangzhou, China, among the office workers who operate the 976 computer workstations in the 32 different departments and units of the Guangzhou Power Supply Bureau, Guangdong Province, China. All participants underwent a comprehensive ocular examination, which included best-corrected visual acuity, intraocular pressure (IOP) measurement by noncontact tonometer (TX-20; Canon Inc. Ltd., Tokyo, Japan), and slit-lamp anterior segment examination by noncycloplegic, A-scan ultrasound biometry (IOL Master; Carl Zeiss Meditec AG, Jena, Germany). The findings of fundus examinations, including posterior pole and peripheral retinal lesions, were recorded.

The study was approved by the Institutional Review Board of Zhongshan Ophthalmic Center and adhered to the tenets of the Declaration of Helsinki. Written informed consent was obtained from all subjects.

### 2.2. Assessment of Refractive Error and Axial Length

Each worker's refractive error was obtained with an autorefractor machine (AR-330A/310A; Nidek Co., Ltd., Beautshire, Japan). Subsequently, subjective refraction was determined by trained, certified study optometrists to achieve best-corrected visual acuity. The final subjective refraction result was used in the analysis. Spherical equivalent (SE) was defined as a sphere plus a half negative cylinder. Axial length (AL) was measured using noncontact partial coherence laser interferometry (IOL Master; Carl Zeiss Meditec AG, Jena, Germany).

### 2.3. Assessment of Peripheral Retinopathy

All subjects were imaged with a scanning laser ophthalmoscope (SLO; Optos, Daytona, United Kingdom). If any suspicious peripheral retinopathy from the SLO was found, the observation from the dilated fundus examination with slit-lamp microscopy, combined with the three-mirror contact lens, served as the standard, and this was recorded on a predesigned form. The dilated pupil size was at least 6 mm, measured using a Haab's pupillometer. Standard digital fundus photography for the central fundus was performed for all subjects by the same observer.

### 2.4. Statistical Analysis

The data were processed and analyzed statistically using STATA software (version 14.0; STATA Corp., College Station, TX). Data were expressed as the mean (SD). Demographic data and clinical measurements were tabulated for all participants and by gender. The significance of gender differences was determined using an independent sample *t*-test when the data were normally distributed, or with the Mann–Whitney *U* test when the data showed a nonparametric distribution. The significance of differences among different age groups was determined using analysis of variance (ANOVA) or the Kruskal–Wallis test. Univariate and multivariate logistic regressions were used to identify potential participant characteristics that were associated with retinal changes. The associated factors were evaluated using a generalized estimating equation model, taking into consideration the correlation structure between both eyes of each subject. For all the tests, *p* < 0.05 was considered statistically significant. The difference and bilateral test formulas were used to determine the minimum sample size.

## 3. Results

In this study, we included the 1934 eyes of 967 subjects (513 females and 454 males) with a mean age of 37.1 (8.3) years (range, 21 to 59 years). The mean uncorrected visual acuity of all subjects was 0.55 (0.44) (median, 0.40; range, 0.02 to 1.5), while the mean corrected visual acuity of all subjects was 1.16 (0.15) (median, 1.2; range, 0.1 to 1.5). The mean AL was 24.63 (1.40) mm, and the mean SE was −2.20 (2.55) D (median, −1.75; range, −15.0 to 4.5). Myopia was found in 68.9% of eyes. The mean computer use time was 8.0 (1.9) hours. No significant gender differences were noted for age, UCVA, BCVA, IOP, SE, C/D, computer use time, and working years. As expected, the AL was significantly shorter in females than in males (*p* < 0.001). Details of the demographic and clinical data of the study population are shown in Table 1.

### 3.1. Frequencies of Retinal Changes

The frequencies of retinal changes found are shown in Table 2. Previous studies classified various changes as myopia-related retinal changes, and this classification was adopted in this study [8, 9]. Most eyes had optic nerve crescents (81.3%), and the white-without-pressure (WWOP) was 15.5%. Various peripheral degenerations were found: 7 eyes (0.4%) had microcystoid degeneration, 40 (2.1%) had peripheral pigmentary degeneration, 87 (4.5%) had lattice degeneration, and 4 (0.2%) had snail-track degeneration. The position of peripheral degenerations was mostly at TT (46.0%), followed by TS (33.3%), NN (16.1%), and NS (4.6%). In total, 19 (1.0%) eyes had paving-stone degeneration, 11 (0.6%) had a retinal hole or tear, and 16 (0.8%) had chorioretinal degeneration. In summary, 374 (19.3%) eyes had some type of peripheral retinal change.

### 3.2. Clinical Characteristics among Different Age Subgroups

Table 3 shows a comparison of the clinical characteristics across different ages. Subjects aged 20–30 years had the lowest UCVA (0.42 (0.42), *p* < 0.001), while subjects aged 50–60 years had the lowest BCVA (1.08 (0.22), *p* < 0.001). In total, 79.1% of eyes were myopic in subjects aged 20–29 years, 72.9% in subjects aged 30–39 years, 62.2% in subjects aged 40–49 years, and 43.4% in subjects aged 50–59 years; these differences were statistically significant (*p* < 0.001). The mean SE in the different subgroups was −3.02 (2.67) D in subjects aged 20–29 years, −2.33 (2.46) D in those aged 30–39 years, −1.60 (2.15) D in those aged 40–49 years, and −1.44 (3.29) D in those aged 50–59 years (*p* < 0.001). As would be expected, subjects aged 20–30 years had the greatest AL (*p* < 0.001) and the largest optic nerve crescent (*p* < 0.001), followed by those aged 30–39 years, 40–49 years, and 50–59 years. The mean computer use time per day was longer in those aged 20–29 years than in the others (*p* < 0.001). Conversely, 120 eyes (27.3%) in those aged 20–29 years showed peripheral retinal changes, as did 143 eyes (18.7%) in those aged 30–39 years, 84 eyes (14.3%) in those aged 40–49 years, and 27 eyes (19.4%) in those aged 50–59 years; these differences among the age groups were also statistically significant (*p* < 0.001).

### 3.3. Association between Retinal Changes and Potential Risk Factors

The analysis of potential risk factors included consideration of a possible relationship between retinal changes and age, gender, AL, SE, IOP, C/D, optic nerve crescent, and the mean computer use time per day. Logistic regression was conducted to assess whether these factors significantly predicted the existence of any peripheral retinal changes. The odds ratios are summarized in Table 4. In the univariate regression analysis, the odds ratio suggested that any peripheral retinal changes were associated with younger age (OR: 0.97 (0.95, 0.98), *p* < 0.001), greater AL (OR: 1.54 (1.42, 1.68), *p* < 0.001), and more serious SE (OR: 0.76 (0.73, 0.80), *p* < 0.001). When these three predictor variables were considered together in multivariate regression, only a greater AL (OR: 1.18 (1.03, 1.35), *p*=0.012) and a more serious SE (OR: 0.82 (0.77, 0.88), *p* < 0.001) were significant risk factors for peripheral retinal changes.

## 4. Discussion

### 4.1. Prevalence of High Myopia-Related Retinal Changes

To the best of our knowledge, this study is the first to show explicitly the prevalence of retinal changes in office computer workers. We included the 1934 eyes of 967 subjects. The ALs were longer in male than in female workers, but no other statistically significant gender differences were noted for age, UCVA, BCVA, IOP, SE, C/D, computer use times, and working years (Table 1). These results are in accordance with previously reported data in the literature [10], especially in populations from the United States [11, 12], Germany [13], Australia [14], and Iceland [15].

Overall, 68.9% of the subjects in our study were myopic, and this prevalence was higher than previously reported in other studies. However, this may reflect the fact that the majority of the population in Guangzhou is ethnic Chinese and that the prevalence of myopia among this urban Chinese population is one of the highest in the world [16]. Interestingly, myopia is occurring at a younger age: 79.1% of the eyes were myopic in subjects aged 20–29 years, 72.9% in those aged 30–39 years, 62.2% in those aged 40–49 years, and 43.4% in those aged 50–59 years.

This higher prevalence of myopia prompted us to adopt the parameters used in previous studies that classified various changes as myopia-related retinal changes [17, 18]. We found an optic nerve crescent (beta peripapillary atrophy) in 81.3% of the subjects' eyes. Many studies have been conducted to examine the prevalence of myopia-related retinal degeneration [19]. A high prevalence of optic nerve crescents was reported in myopic adolescents by Samarawickrama et al., who found an optic nerve crescent in 92% of eyes with myopia [20]. Again, this may be related to the sample size and the difference in subject populations, since the previous researchers recruited only a myopic population, whereas our study was only concerned with office computer workers [20]. Our results also demonstrated a lower prevalence of retinal changes when compared to previous studies that examined myopic populations. We found that more than 19.3% of our subjects had one or more peripheral retinal lesions, and that 0.8% of our subjects had a posterior pole chorioretinal lesion. However, these ratios are in accordance with previous studies.

The peripheral retinal lesion noted most frequently was the WWOP type [21–23]. Peripheral retinal degenerative lesions, such as lattice degeneration and retinal holes or breaks, which are considered important risk factors for retinal detachment, were found in 4.6% and 0.6% of eyes, respectively, and the detectable rate of degenerative lesions in the peripheral retina was found more frequently in the temporal location than in the nasal location [17, 24]. The highest number were located in the inferior temporal quadrant (46%), followed by the superior temporal quadrant (33.3%), and inferior nasal quadrant (16.1%), with the lowest number in the superior nasal quadrant (4.6%) (Table 2).

Age did not appear to have a major influence, although it could be a factor affecting prevalence, as peripheral retinal changes were less frequently found in our subjects who were more than 50 years old (Table 3), as documented previously [25–29]. Our study also confirmed that the eyesight of the naked eye is better in older people than in younger people, which may be due to serious myopic problems in younger people. As yet, we do not know what accounts for this. However, the following two possibilities have occurred to us. First, this result could reflect an increase in the prevalence of myopia in younger and younger people, together with increased computer use, which has had the greatest impact on our lives in modern times [6, 30, 31]. Studies have shown an association between increased AL and the severity of myopia with peripheral retinal degeneration and an increased prevalence of peripheral retinal degeneration in myopia [17, 32–35]. The present study demonstrated that the incidence of myopia is higher in younger adults. A second possibility is that length of time using a computer declines as people age. Worldwide estimates indicate that 25% of computer users are already suffering from computer-related injuries. Therefore, we find it likely that eye injuries are also possible, because (1) reading and visual work when using a computer leads to a change in the shape of the cornea, which may lead to the development of myopia [31]; (2) there is a significant positive association between playing video games for more than four hours per week and progression of myopia [31]. However, when comparing subjects aged 40–49 and 50–59, peripheral retinal degeneration was more prevalent in the older group. One of the most important reasons for the conflicting results in these studies may be the increased incidence of peripheral retinal atrophy in the elderly.

It is difficult to ascertain on the basis of previous studies how myopia alone affects the retina. Therefore, we tried to reduce the age effect on retinal degeneration by the use of multivariate regression to determine myopia-related retinal changes. However, the multivariate regression revealed only greater AL and more serious SE as potential risk factors for retinal changes (Table 4). This may be related to the greater prevalence of myopia in young people than in the elderly. Having reviewed the literature, Saw and colleagues suggested that chorioretinal abnormalities such as lattice degeneration are associated with refractive error and AL. This agrees with our findings, which showed that eyes with peripheral retinal degeneration had greater AL and more serious SE than did eyes without peripheral retinal degeneration.

The aim of the present study was to determine the prevalence of peripheral retinal changes in ethnic Chinese office computer workers in Guangzhou. Our findings raise one important point: All participants were from the Guangzhou Power Supply Bureau, and most of them were senior intellectuals—a group with a known association with a high incidence of high myopia—and previous studies have reported peripheral retinal degenerative lesions in a considerable proportion of subjects with high myopia. As the myopic young adult population is at high risk of developing pathological myopia, we appeal for publications to pay more attention to the fundus health of young people, especially those with myopia. Health behaviour programs aimed at increasing outdoor time may also help to prevent incident myopia or to slow its progression.

Future research in this area should consider several issues, including adequate subject numbers and subjects from different departments. Our sample size was 967 subjects from 32 different departments, which was greater than our minimum calculated sample size (766).

## 5. Conclusion

Peripheral retinal degenerative changes and optic nerve crescents were found in a significant proportion of our subjects, who perform computer work in an office. Of these retinal changes, 0.6% were sight-threatening changes, and 19.3% were peripheral retinal changes. Myopia is occurring in younger and younger people, accompanied by peripheral retinal degeneration. With the modernization of society, we should pay more attention to the issue of myopia among young people.

## Figures and Tables

**Table 1:** Clinical characteristics of the study subjects.

Characteristic	Overall	Gender		*p* value
		Female	Male	
Number of patients (%)				
Total	967 (100)	513 (53.1)	454 (46.9)	—
20–30 y	220 (22.8)	112 (11.6)	108 (11.2)	—
30–39 y	383 (39.6)	200 (20.7)	183 (18.9)	—
40–49 y	295 (30.5)	178 (18.4)	117 (12.1)	—
50–59 y	69 (7.1)	24 (2.4)	45 (4.7)	—
Age (y)	37.1 (8.3)	37.2 (8.0)	37.0 (8.7)	0.758
UCVA	0.55 (0.44)	0.55 (0.44)	0.55 (0.45)	0.753
BCVA	1.16 (0.15)	1.15 (0.14)	1.16 (0.16)	0.193
IOP (mmHg)	14.2 (2.7)	14.1 (2.7)	14.3 (2.8)	0.089
Al (mm)	24.63 (1.40)	24.37 (1.29)	24.92 (1.46)	**<0.001**
SE (D)	−2.20 (2.55)	−2.19 (2.47)	−2.21 (2.63)	0.845
Number of myopia eyes (%)	1334 (68.9)	715 (69.6)	619 (68.1)	0.567
C/D	0.32 (0.11)	0.32 (0.11)	0.33 (0.11)	0.155
Work hours per day (h)	8.0 (1.9)	8.1 (1.5)	8.0 (2.2)	0.403
Work years (y)	13.7 (8.9)	14.3 (8.8)	13.6 (9.2)	0.281
Optic nerve crescent	0.29 (0.29)	0.28 (0.28)	0.30 (0.30)	0.265

Data are expressed as the mean (SD); UCVA = uncorrected visual acuity; BCVA = best-corrected visual acuity; IOP = intraocular pressure; AL = axial length; SE = spherical equivalent; D = diopter; C/D = cup/disc ratio.

**Table 2:** Frequencies of myopia-related retinal changes.

Retinal change	Number of eyes	Percentage (%)
Optic nerve crescent	1574	81.3
WWOP	300	15.5
Microcystoid degeneration	7	0.4
Peripheral pigmentary degeneration	40	2.1
Lattice degeneration	87	4.6
Snail-track degeneration	4	0.2
Any degeneration position		
NS	6	4.6
NN	22	16.1
TS	45	33.3
TT	63	46.0
Paving-stone degeneration	19	1.0
Retinal hole or tear	11	0.6
Chorioretinal degeneration	16	0.8
Retinal detachment	0	0
Any peripheral retinal change	374	19.3

**Table 3:** Comparision of clinical characteristics among different age subgroups.

	20–30 y	30–39 y	40–49 y	50–59 y	*p* value
UCVA	0.42 (0.42)	0.54 (0.44)	0.64 (0.45)	0.65 (0.43)	<0.001
BCVA	1.16 (0.15)	1.17 (0.14)	1.14 (0.14)	1.08 (0.22)	<0.001
Al (mm)	25.06 (1.40)	24.66 (1.32)	24.32 (1.32)	24.36 (1.76)	<0.001
SE (D)	−3.02 (2.67)	−2.33 (2.46)	−1.60 (2.15)	−1.44 (3.29)	<0.001
Number of myopia eyes (%)	348 (79.1)	559 (72.9)	367 (62.2)	60 (43.4)	<0.001
C/D	0.32 (0.10)	0.32 (0.11)	0.34 (0.11)	0.35 (0.12)	0.001
Optic nerve crescent	0.34 (0.28)	0.29 (0.27)	0.24 (0.29)	0.26 (0.35)	<0.001
Computer hours per day (h)	8.4 (1.6)	8.2 (1.7)	7.7 (2.0)	7.1 (2.5)	<0.001
Any peripheral retinal change	120 (27.3)	143 (18.7)	84 (14.3)	27 (19.4)	<0.001

**Table 4:** Uni- and multivariate logistic regression for association between retinal changes and potential risk factors.

	Univariate		Multivariate	
	OR (95% CI)	*p* value	OR (95% CI)	*p* value
Age	0.97 (0.95, 0.98)	<0.001	0.98 (0.97, 1.00)	0.144
Gender	1.04 (0.83, 1.30)	0.708	—	—
AL	1.54 (1.42, 1.68)	<0.001	1.18 (1.03, 1.35)	0.012
SE	0.76 (0.73, 0.80)	<0.001	0.82 (0.77, 0.88)	<0.001
IOP	1.03 (0.99, 1.07)	0.131	—	—
C/D	0.85 (0.32, 2.30)	0.762	—	—
Computer hours per day	0.96 (0.91, 1.03)	0.307	—	—

## Data Availability

All the data related to this article are in the manuscript and are available from the corresponding author on reasonable request.

## Supplementary Materials

Supplementary 1 Picture 1: lattice degeneration.

Supplementary 2 Picture 2: lattice degeneration and retinal hole.

Supplementary 3 Picture 3: retinal tear.

Supplementary 4 Picture 4: microcystoid degeneration.

Supplementary 5 Picture 5: paving-stone degeneration.

Supplementary 6 Picture 6: snail-track degeneration.

Supplementary 7 Picture 7: optic nerve crescents.

Supplementary 8 Picture 8: white-without-pressure (WWOP).

Supplementary 9 Picture 9: pigmentary degeneration (od).

Supplementary 10 Picture 10: pigmentary degeneration (os).

## References

[1] Blehm C., Vishnu S., Khattak A., Mitra S., Yee R. W. (2005). Computer vision syndrome: a review. *Survey of Ophthalmology*.

[2] Klamm J., Tarnow K. G. (2015). Computer vision syndrome: a review of literature. *Medsurg Nursing: Official Journal of the Academy of Medical-Surgical Nurses*.

[3] Woods V. (2005). Musculoskeletal disorders and visual strain in intensive data processing workers. *Occupational Medicine*.

[4] Logaraj M., Madhupriya V., Hegde S. (2014). Computer vision syndrome and associated factors among medical and engineering students in chennai. *Annals of Medical and Health Sciences Research*.

[5] Qiu X., Kumbalasiri T., Carlson S. M. (2005). Induction of photosensitivity by heterologous expression of melanopsin. *Nature*.

[6] Tosini G., Ferguson I., Tsubota K. (2016). Effects of blue light on the circadian system and eye physiology. *Molecular Vision*.

[7] Jomoah I. M. (2014). Work-related health disorders among Saudi computer users. *The Scientific World Journal*.

[8] Cheng S. C., Lam C. S., Yap M. K. (2013). Prevalence of myopia-related retinal changes among 12-18 year old Hong Kong Chinese high myopes. *Ophthalmic and Physiological Optics*.

[9] Bansal A. S., Hubbard G. R. (2010). Peripheral retinal findings in highly myopic children < or =10 years of age. *Retina*.

[10] Gozum N., Çakir M., Gücukoglu A., Sezen F. (1997). Relationship between retinal lesions and axial length, age and sex in high myopia. *European Journal of Ophthalmology*.

[11] Lee K. E., Klein B. E., Klein R., Quandt Z., Wong T. Y. (2009). Association of age, stature, and education with ocular dimensions in an older white population. *Archives of Ophthalmology*.

[12] Jivrajka R., Shammas M. C., Boenzi T., Swearingen M., John Shammas H. (2008). Variability of axial length, anterior chamber depth, and lens thickness in the cataractous eye. *Journal of Cataract & Refractive Surgery*.

[13] Hoffmann P. C., Hutz W. W. (2010). Analysis of biometry and prevalence data for corneal astigmatism in 23,239 eyes. *Journal of Cataract & Refractive Surgery*.

[14] Fotedar R., Wang J. J., Burlutsky G. (2010). Distribution of axial length and ocular biometry measured using partial coherence laser interferometry (IOL Master) in an older white population. *Ophthalmology*.

[15] Olsen T., Arnarsson A., Sasaki H., Sasaki K., Jonasson F. (2007). On the ocular refractive components: the Reykjavik eye study. *Acta Ophthalmologica Scandinavica*.

[16] Fan D. S., Lam D. S. C., Lam R. F. (2004). Prevalence, incidence, and progression of myopia of school children in Hong Kong. *Investigative Opthalmology & Visual Science*.

[17] Lam D. S., Fan D. S. P., Chan W. M. (2005). Prevalence and characteristics of peripheral retinal degeneration in Chinese adults with high myopia: a cross-sectional prevalence survey. *Optometry and Vision Science*.

[18] Liu H. H., Xu L., Wang Y. X., Wang S., You Q. S., Jonas J. B. (2010). Prevalence and progression of myopic retinopathy in Chinese adults: the Beijing eye study. *Ophthalmology*.

[19] Lai T. Y., Fan D. S. P., Lai W. W. K., Lam D. S. C. (2008). Peripheral and posterior pole retinal lesions in association with high myopia: a cross-sectional community-based study in Hong Kong. *Eye*.

[20] Samarawickrama C., Mitchell P., Tong L. (2011). Myopia-related optic disc and retinal changes in adolescent children from singapore. *Ophthalmology*.

[21] Shukla M., Ahuja O. P. (1982). White with pressure (WWP) and white without pressure (WWOP) lesions. *Indian Journal of Ophthalmology*.

[22] Freeman H. M. (1978). Fellow eyes of giant retinal breaks. *Transactions of the American Ophthalmological Society*.

[23] Rutnin U., Schepens C. L. (1967). Fundus appearance in normal eyes. III. Peripheral degenerations. *American Journal of Ophthalmology*.

[24] Wilkinson C. P. (2014). Interventions for asymptomatic retinal breaks and lattice degeneration for preventing retinal detachment. *Cochrane Database of Systematic Reviews*.

[25] Gonzales C. R., Gupta A., Schwartz S. D., Kreiger A. E. (2004). The fellow eye of patients with phakic rhegmatogenous retinal detachment from atrophic holes of lattice degeneration without posterior vitreous detachment. *British Journal of Ophthalmology*.

[26] Straatsma B. R., Zeegen P. D., Foos R. Y., Feman S. S., Shabo A. L. (1974). Lattice degeneration of the retina. XXX Edward Jackson memorial lecture. *American Journal of Ophthalmology*.

[27] Byer N. E. (1965). Clinical study of lattice degeneration of the retina. *Transactions–American Academy of Ophthalmology and Otolaryngology*.

[28] Shiomi Y. (1981). Study of lattice degeneration of the retina. Part 2. Clinical features of lattice degeneration of the retina. *Nippon Ganka Gakkai Zasshi*.

[29] Karlin D. B., Curtin B. J. (1976). Peripheral chorioretinal lesions and axial length of the myopic eye. *American Journal of Ophthalmology*.

[30] O’Hagan J. B., Khazova M., Price L. L. (2016). Low-energy light bulbs, computers, tablets and the blue light hazard. *Eye*.

[31] Saxena R., Vashist P., Tandon R. (2017). Incidence and progression of myopia and associated factors in urban school children in Delhi: the North India Myopia study (NIM study). *PLoS One*.

[32] Hyams S. W., Neumann E. (1969). Peripheral retina in myopia. With particular reference to retinal breaks. *British Journal of Ophthalmology*.

[33] Celorio J. M., Pruett R. C. (1991). Prevalence of lattice degeneration and its relation to axial length in severe myopia. *American Journal of Ophthalmology*.

[34] Pierro L., Camesasca F. I., Mischi M., Brancato R. (1992). Peripheral retinal changes and axial myopia. *Retina*.

[35] Curtin B. J., Karlin D. B. (1971). Axial length measurements and fundus changes of the myopic eye. *American Journal of Ophthalmology*.

