# Supplementary figures and images for: The Prevalence and Associations of Peripheral Retinopathy: Baseline Study of Guangzhou Office Computer Workers
Source: J Ophthalmol. 2018 Jun 20;2018:2358690. doi: 10.1155/2018/2358690 (PMC6031160; doi:10.1155/2018/2358690)

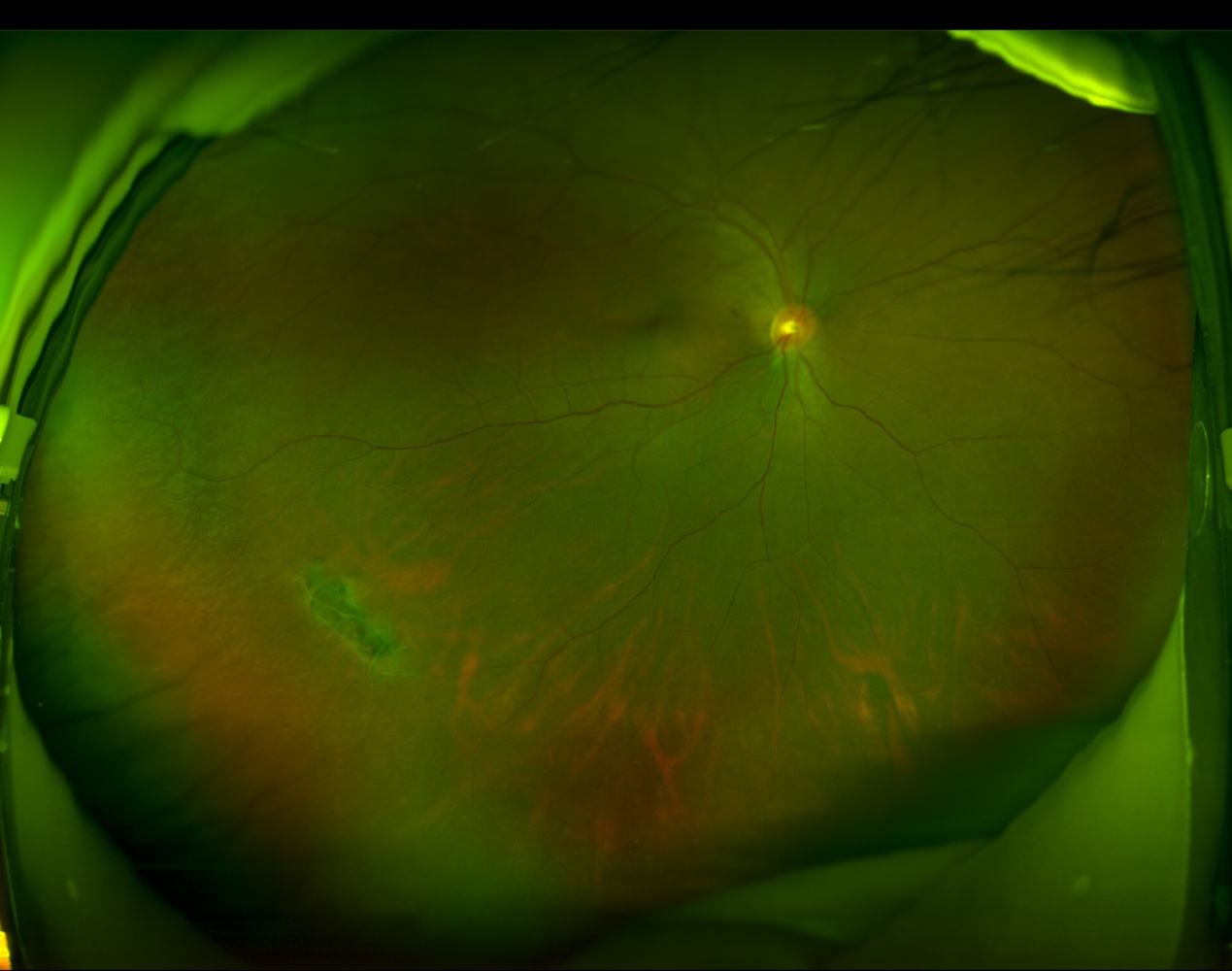


1. Lattice degeneration

Supplement: Supplementary 1 — Picture 1: lattice degeneration. [file 2358690.f1.docx]

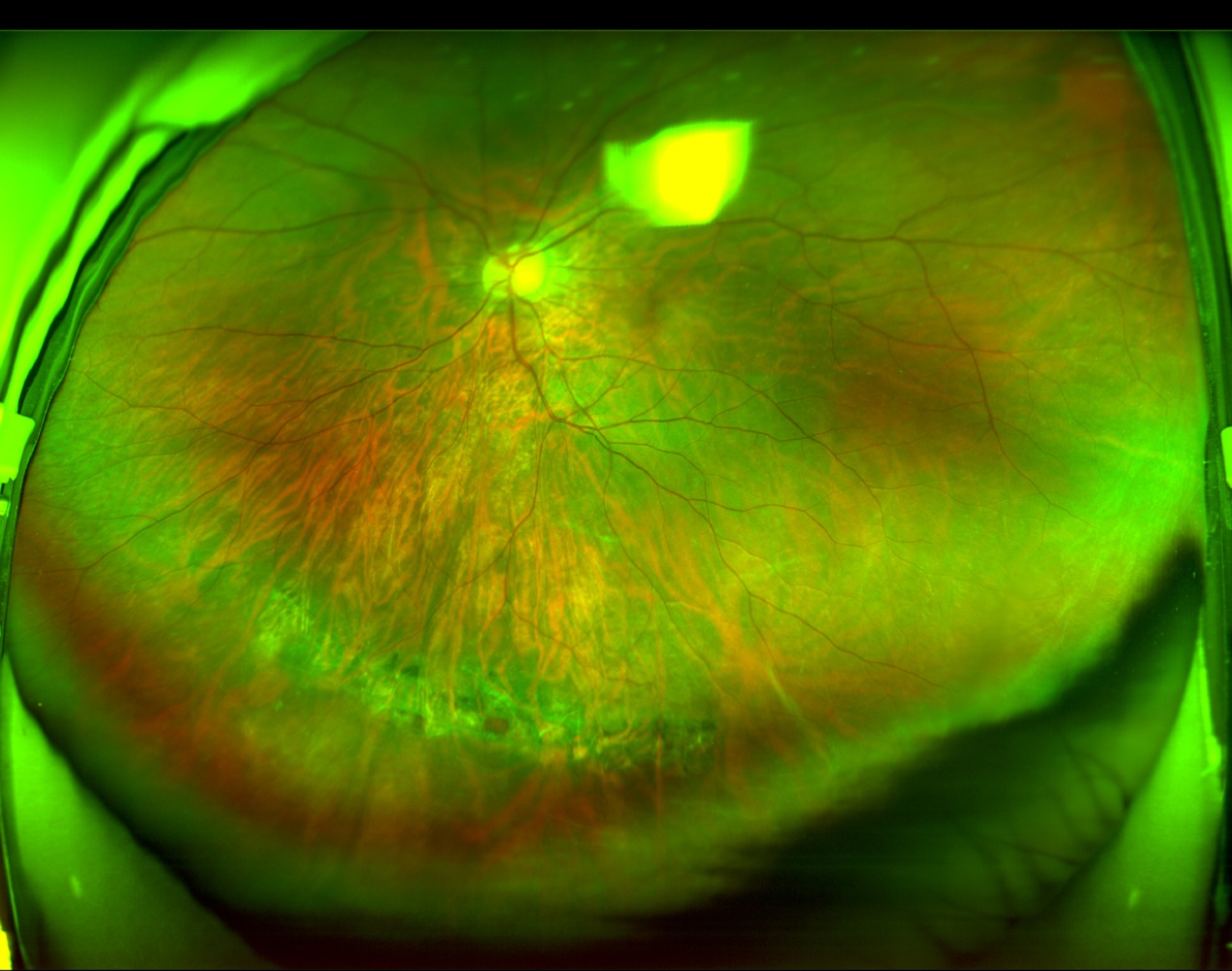


2. Lattice degeneration and retinal hole

Supplement: Supplementary 2 — Picture 2: lattice degeneration and retinal hole. [file 2358690.f2.docx]

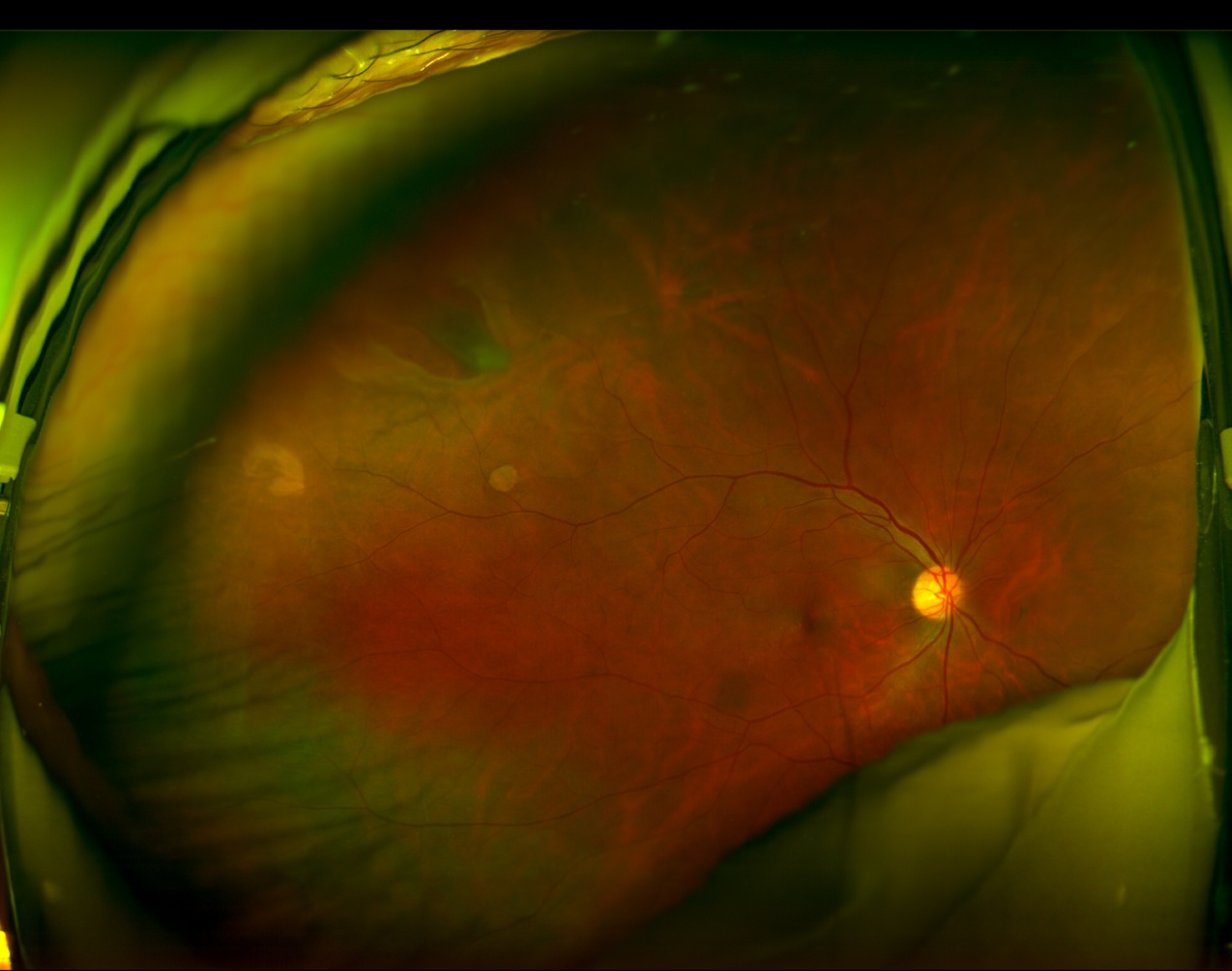


3. Retinal tear

Supplement: Supplementary 3 — Picture 3: retinal tear. [file 2358690.f3.docx]

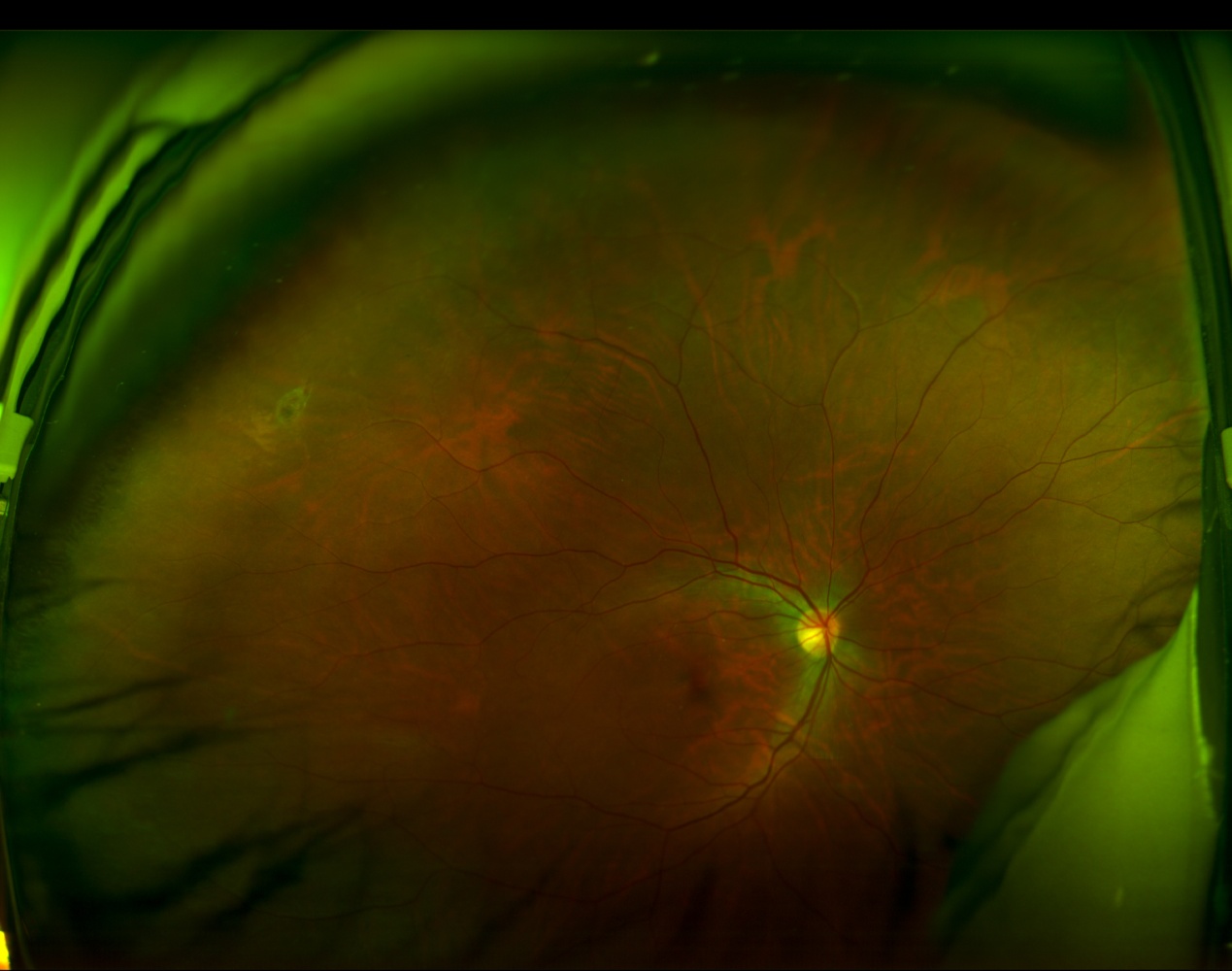


4. Microcystoid degeneration

Supplement: Supplementary 4 — Picture 4: microcystoid degeneration. [file 2358690.f4.docx]

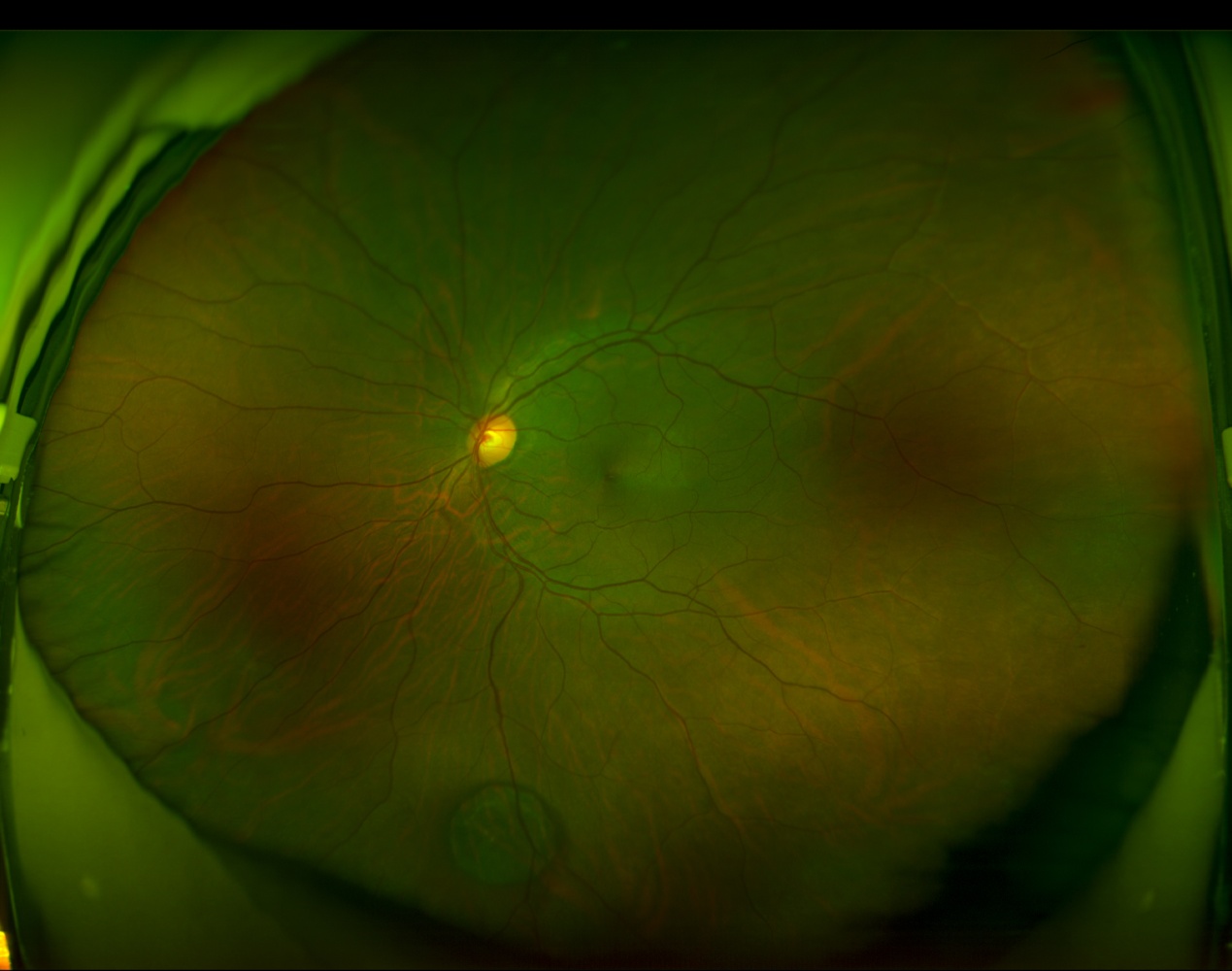


5. Paving-stone degeneration

Supplement: Supplementary 5 — Picture 5: paving-stone degeneration. [file 2358690.f5.docx]

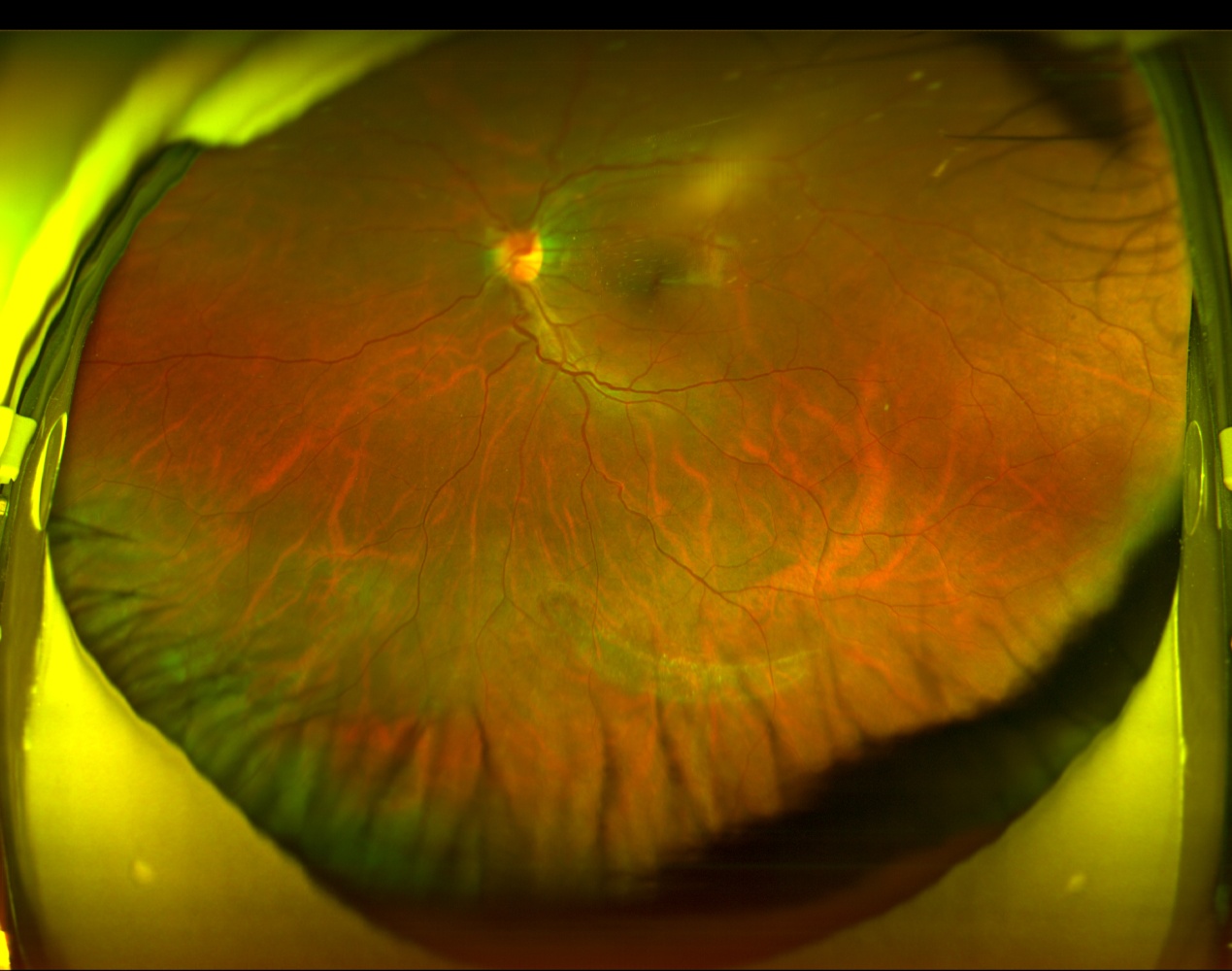


6. Snail-track degeneration

Supplement: Supplementary 6 — Picture 6: snail-track degeneration. [file 2358690.f6.docx]

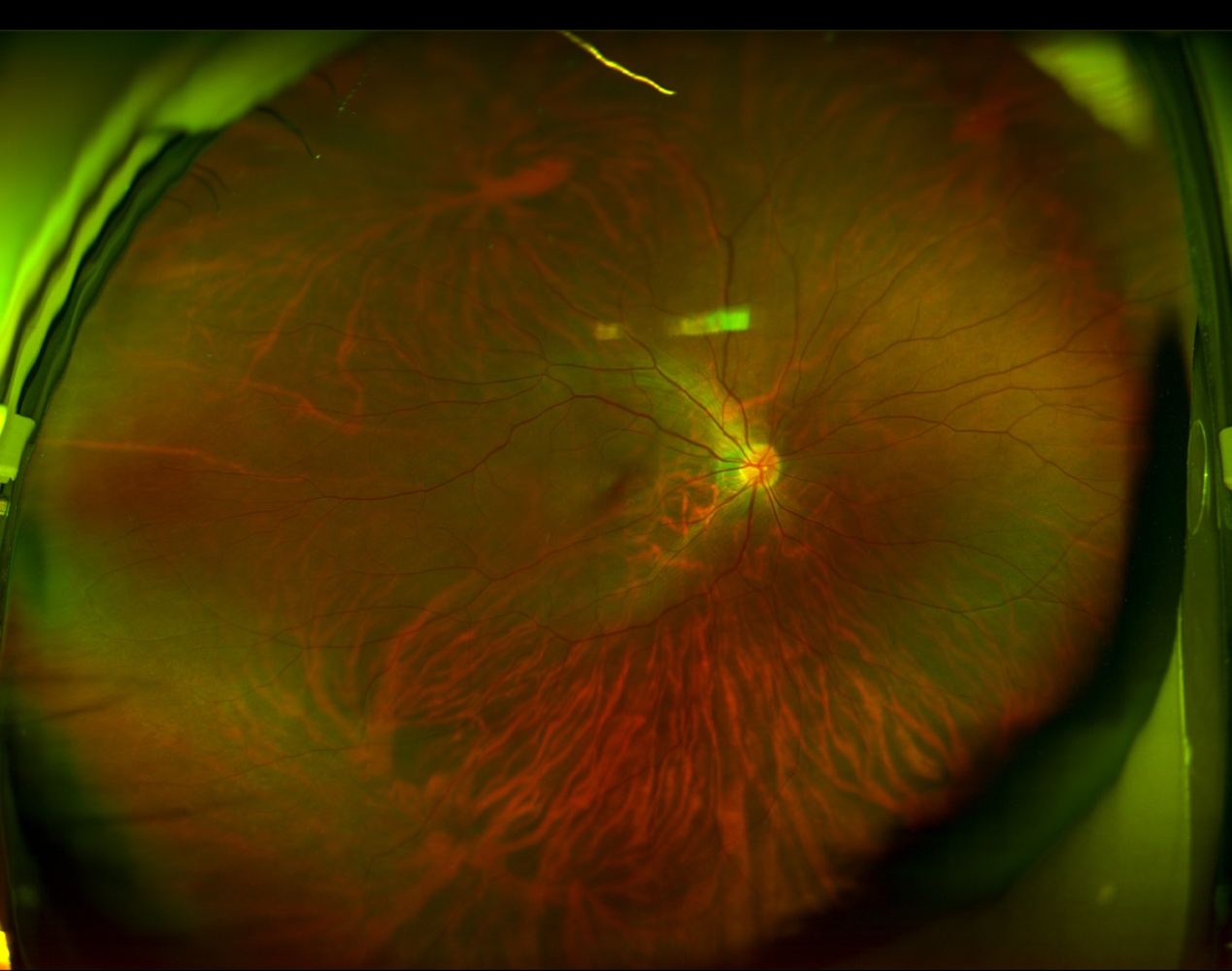


7. Optic nerve crescents

Supplement: Supplementary 7 — Picture 7: optic nerve crescents. [file 2358690.f7.docx]

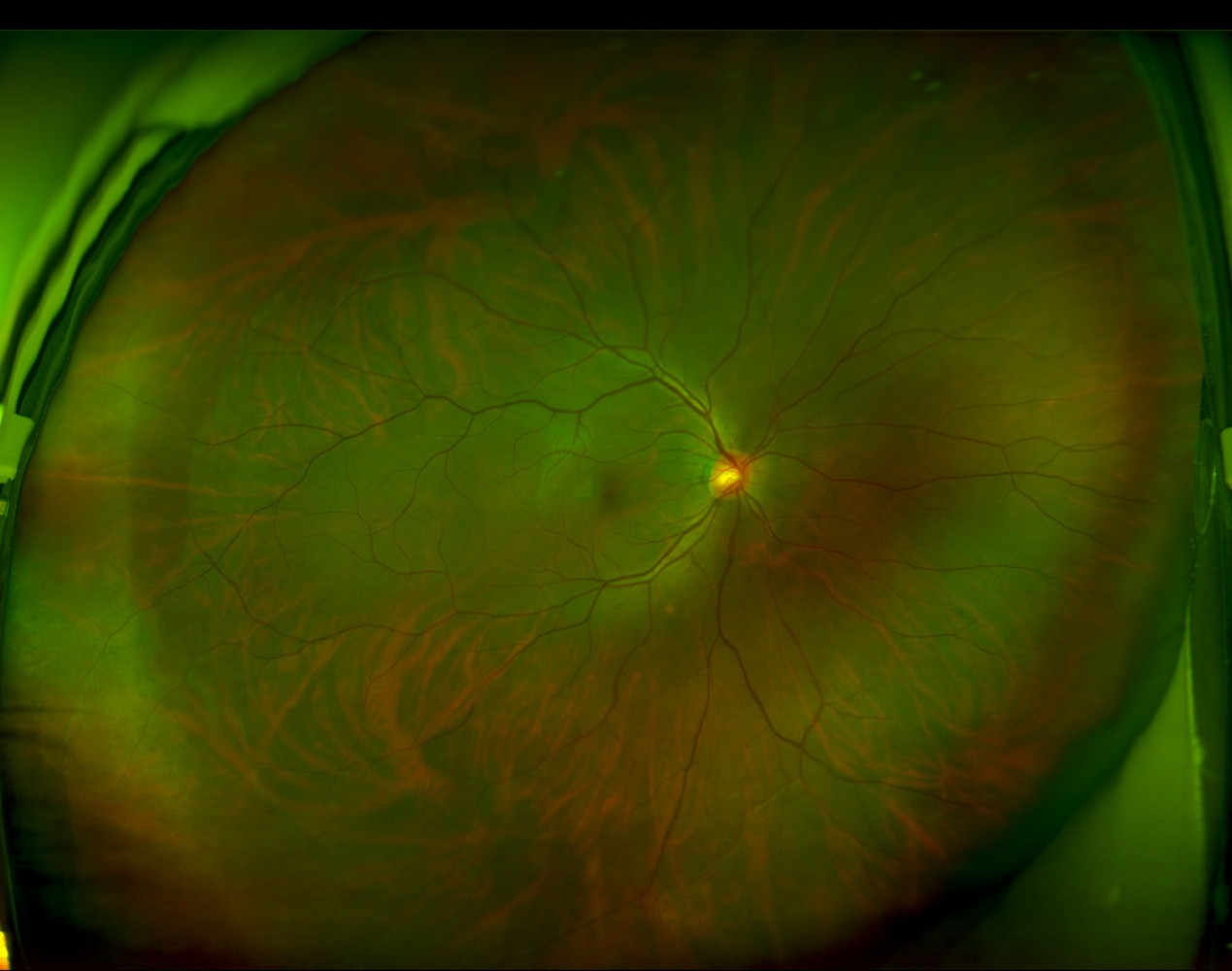


8. White-without-pressure(WWOP)

Supplement: Supplementary 8 — Picture 8: white-without-pressure (WWOP). [file 2358690.f8.docx]

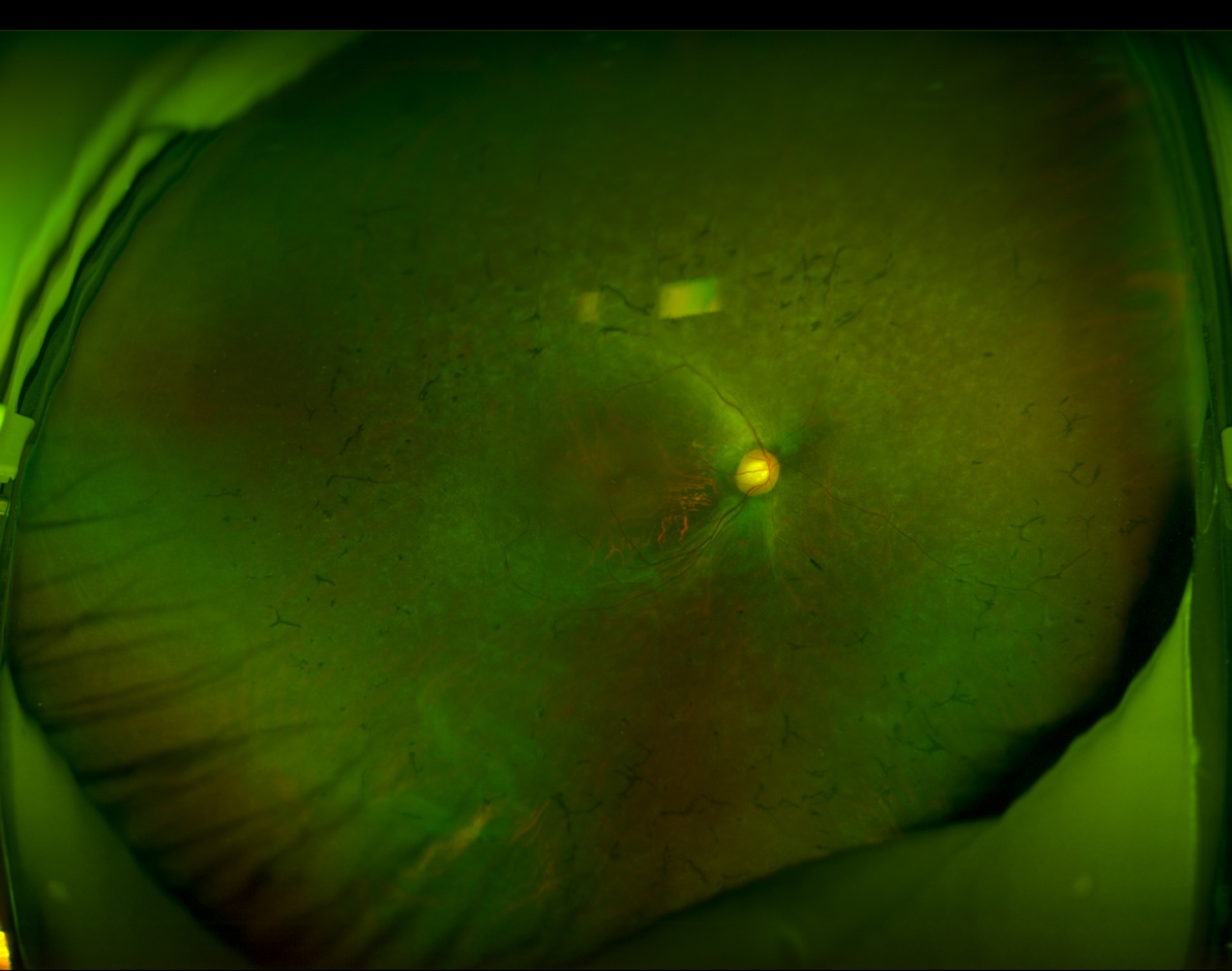


9. Pigmentary degeneration (od)

Supplement: Supplementary 9 — Picture 9: pigmentary degeneration (od). [file 2358690.f9.docx]

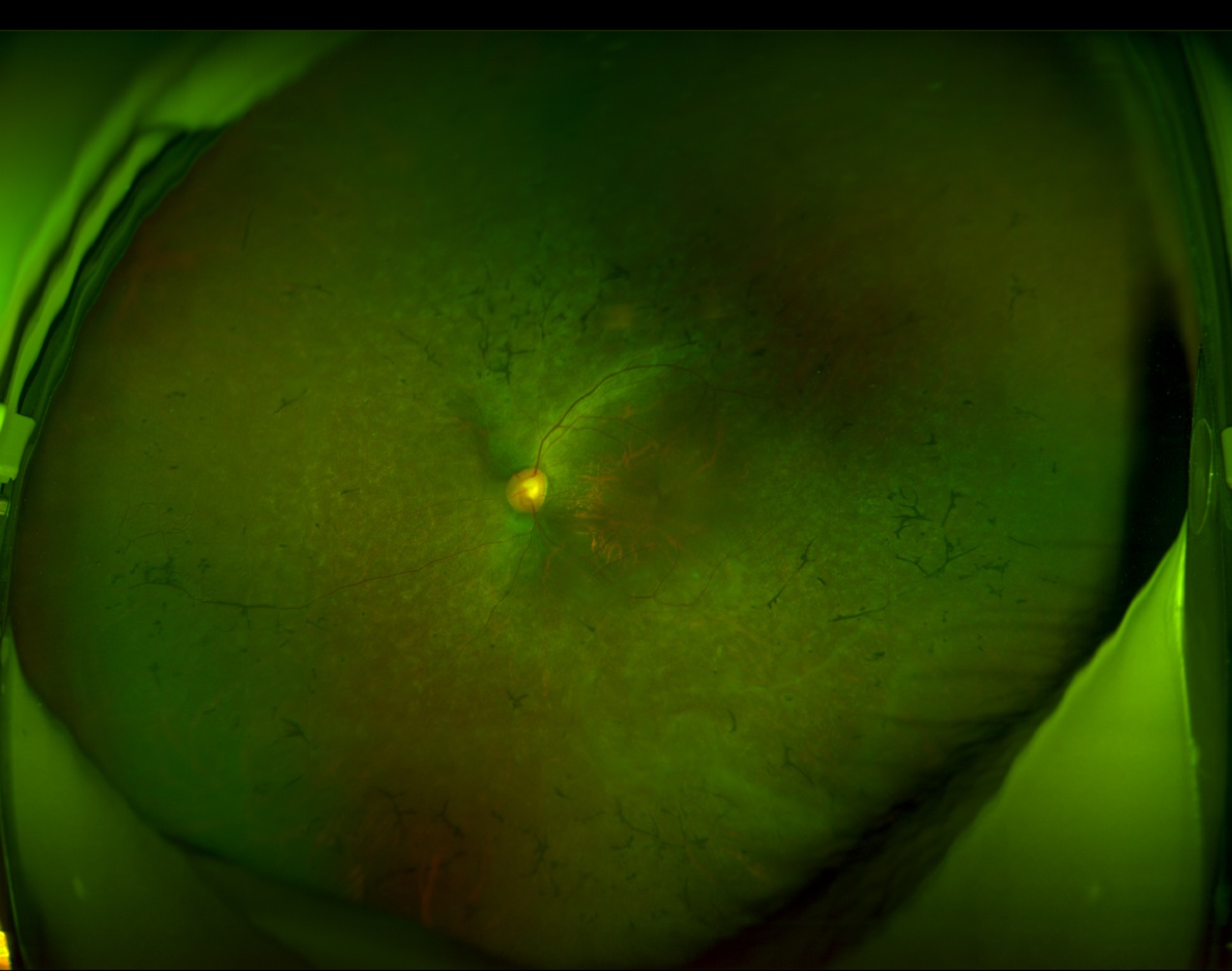


10. Pigmentary degeneration (os)

Supplement: Supplementary 10 — Picture 10: pigmentary degeneration (os). [file 2358690.f10.docx]
